# Supplementary material for: Continuous crystalline graphene papers with gigapascal strength by intercalation modulated plasticization
Source: Nat Commun. 2020 May 27;11:2645. doi: 10.1038/s41467-020-16494-0 (PMC7253461; doi:10.1038/s41467-020-16494-0)
Supplement: Supplementary file 1 — Supplementary Information [file 41467_2020_16494_MOESM1_ESM.pdf]

Supplementary Information

Continuous Crystalline Graphene Papers with Gigapascal Strength by Intercalation Modulated Plasticization

By Li *et al.*

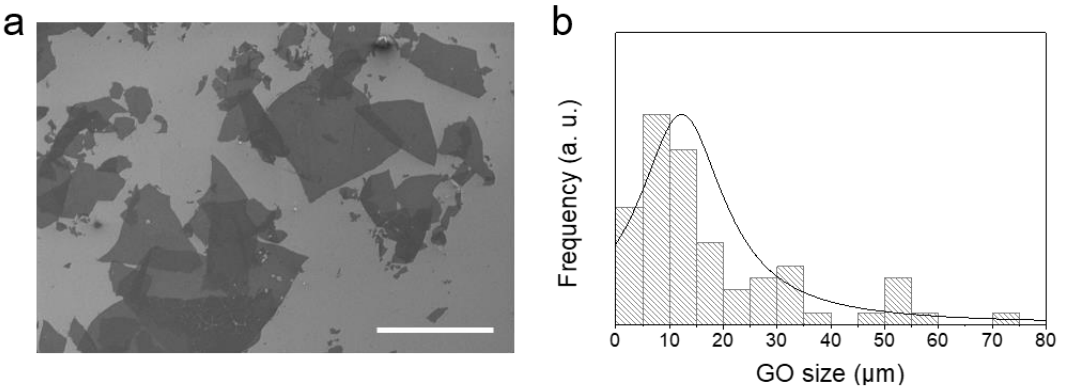

Supplementary Figure 1. Size distribution of GO sheets. **a** and **b,** SEM image (**a**) and corresponding size distribution (**b**) of GO. Scale bar, 50 μm (**a**)

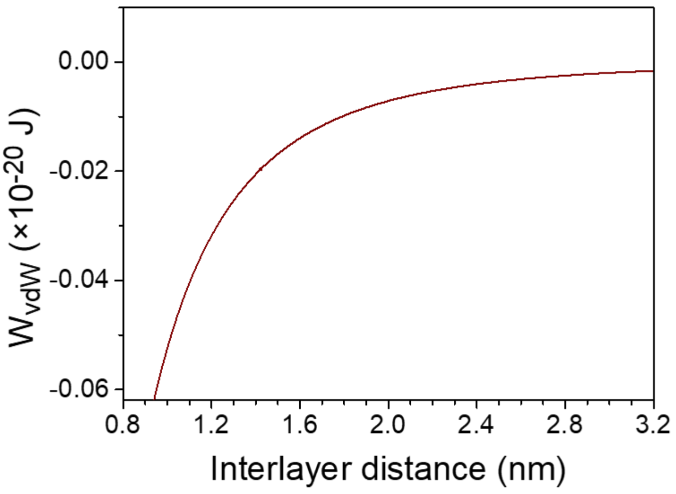

Supplementary Figure 2. The correlation between van der Waals interaction and interlayer distance. As the interlayer distance increases, the van der Waals attraction degrades gradually. The calculation of van der Waals interaction is based on the Lifshiz`s formula as follows:

$$W_{vdW}(d) = -\frac{H}{12\pi}\left(\frac{1}{d^2} + \frac{1}{(d+2t)^2} - \frac{1}{(d+t)^2} - \frac{1}{(d+t)^2}\right) \qquad \text{(Equation 1)}$$

where H is Hamaker constant (3.72×10^{-20} J for ethanol)[1], d is the interlayer distance, and t is the thickness of GO sheet.

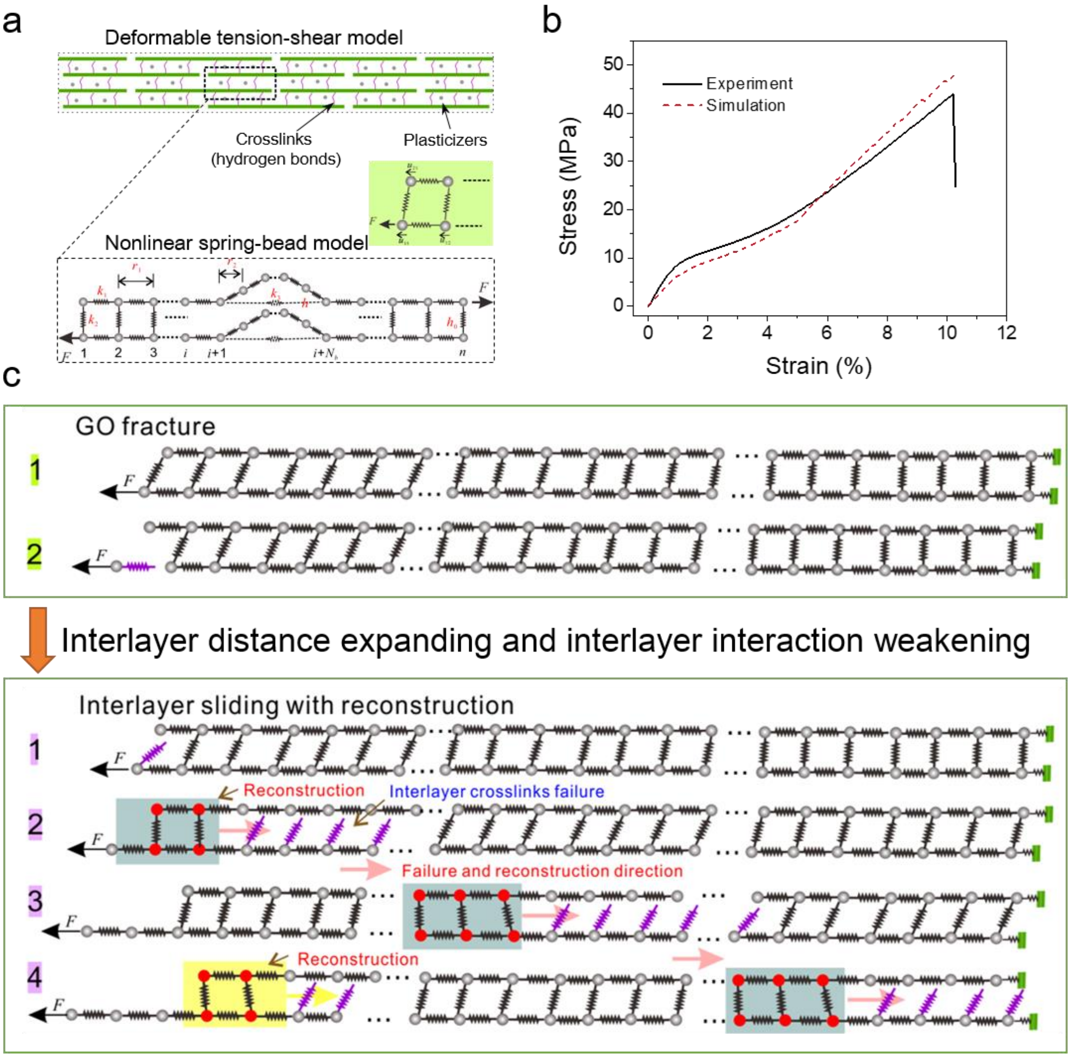

Supplementary Figure 3. Numerical simulation of the intercalation modulated plasticization. **a** Non-linear spring-bead model of the plasticization of GOPs. Here, the two GO sheets are modeled as two chains of beads. The intralayer covalent bonds are simplified as linear springs, while the self-healable interlayer interactions are simplified as the reconstructable springs, and the mass of GO is constricted at the beads. **b** The numerical simulated tensile curve of the plasticized GOP with ethanol intercalated, showing accordance with the experimental result. **c** Schematic of the process of plastic deformation. The interlayer interaction in dried GOPs is powerful that the interlayer sliding is difficult to occur. As the interlayer spacing expands after solvent intercalating, the interlayer sliding is activated, and the failure and reconstruction of the weakening interlayer links continuously occur in balance, contributing to the plastic strain of GOP as the dislocation gliding in metals.

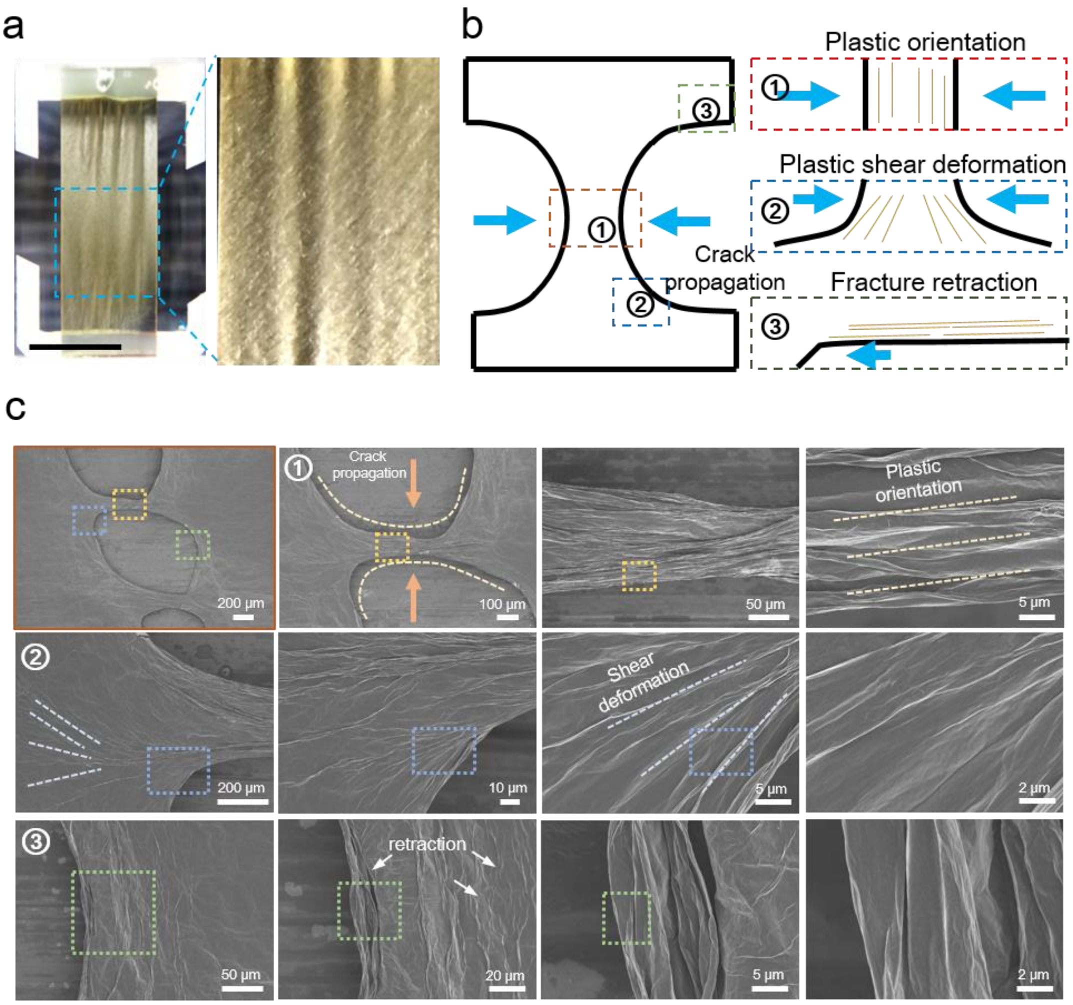

Supplementary Figure 4. Plasticization phenomena of GO papers. **a** Snapshot of plasticized GOPs during tensile test. **b** Schematic of the three typical regimes in plastic deformation. **c** SEM images of the three regimes in plasticized GOPs being about to fracture. Scale bar, 1 mm (**a**)

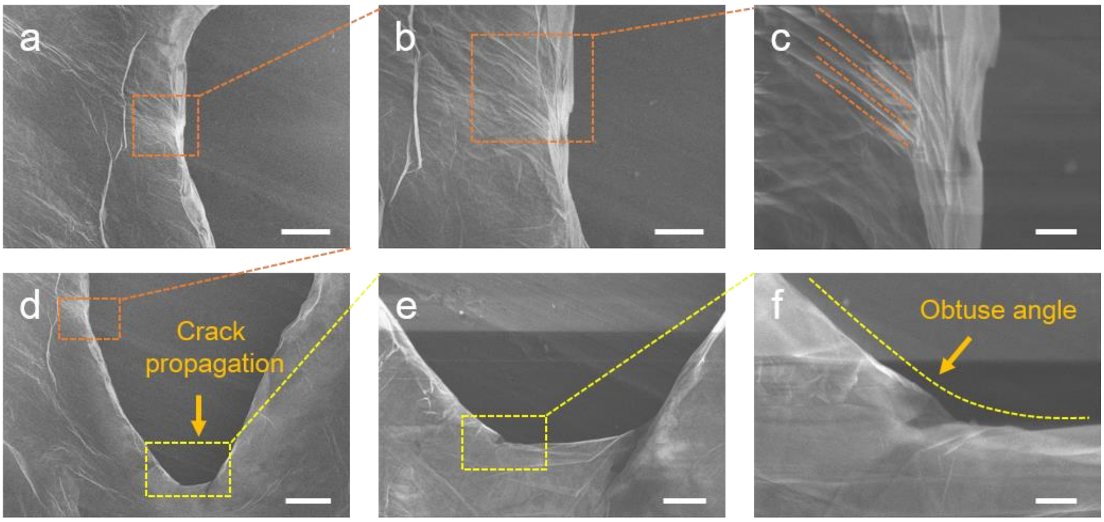

Supplementary Figure 5. The feature of crack propagation in plasticized GO papers. The obtuse slippery propagation without top end indicates the typical plastic fracture. Scale bar, 10 μm (**d**), 5 μm (**a**), 2 μm (**b, e**), and 500 nm (**c, f**)

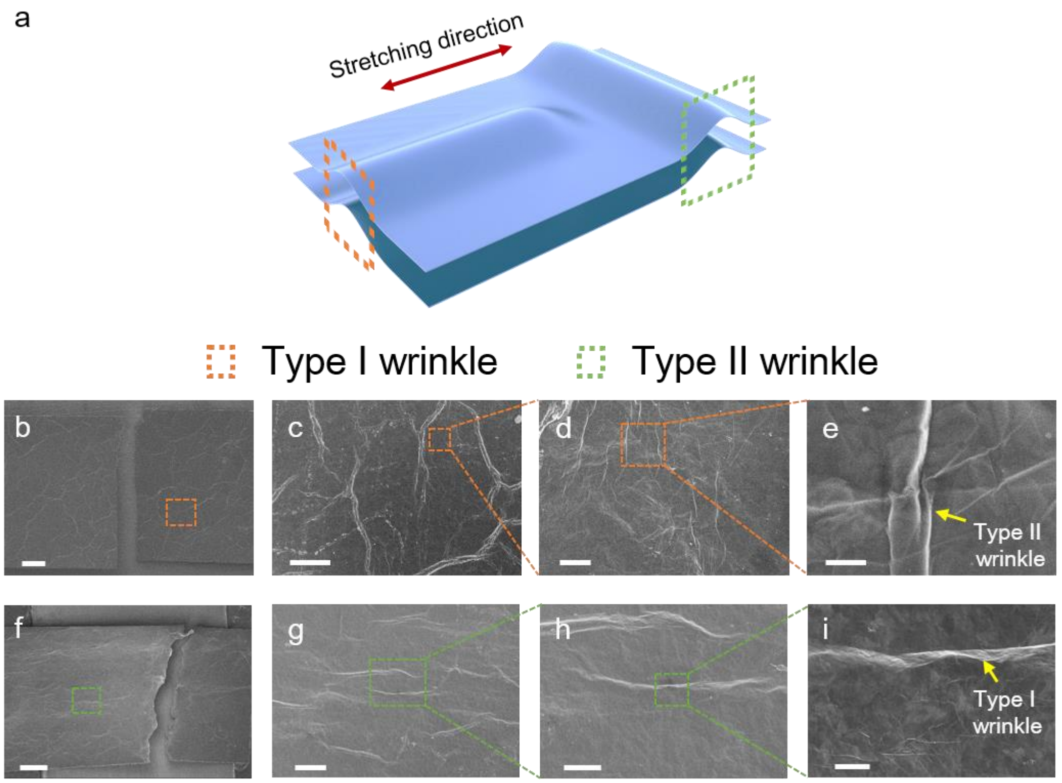

Supplementary Figure 6. The surface feature of fractured GO papers. **a** Schematic of the two types of wrinkles. For clarify, the wrinkles along the stretching direction are defined as type I wrinkles, and the ones perpendicular to the stretching direction are defined as type II wrinkles. **b-e** Surface wrinkled textures of direct-cast GOPs, showing that these wrinkles remain even after elastic breakage. **f-i** Surface features of plasticized GOPs. The gradually emerged aligned texture during IMP stretching suggests that graphene sheets are stretched to extended state. Scale bar, 200 µm (**b, f**), 50 µm (**c, g**), 20 µm (**h**), 5 µm (**d, i**), and 500 nm (**e**)

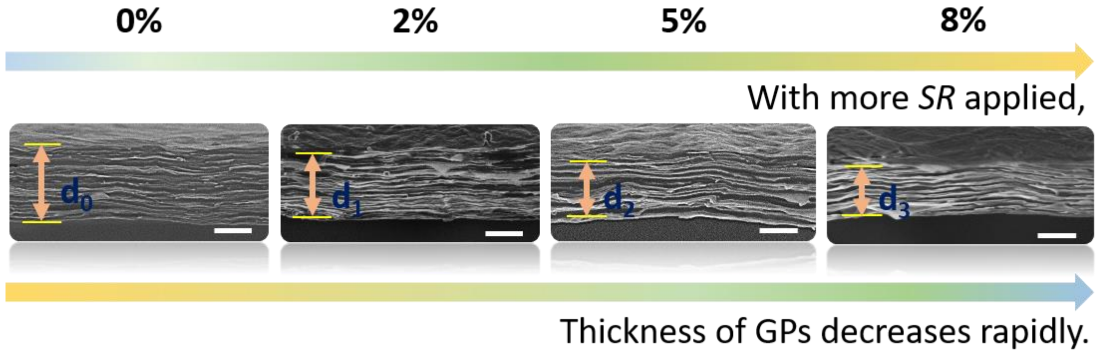

Supplementary Figure 7. Thickness of GPs at different *SR*. Scale bar, 2 μm

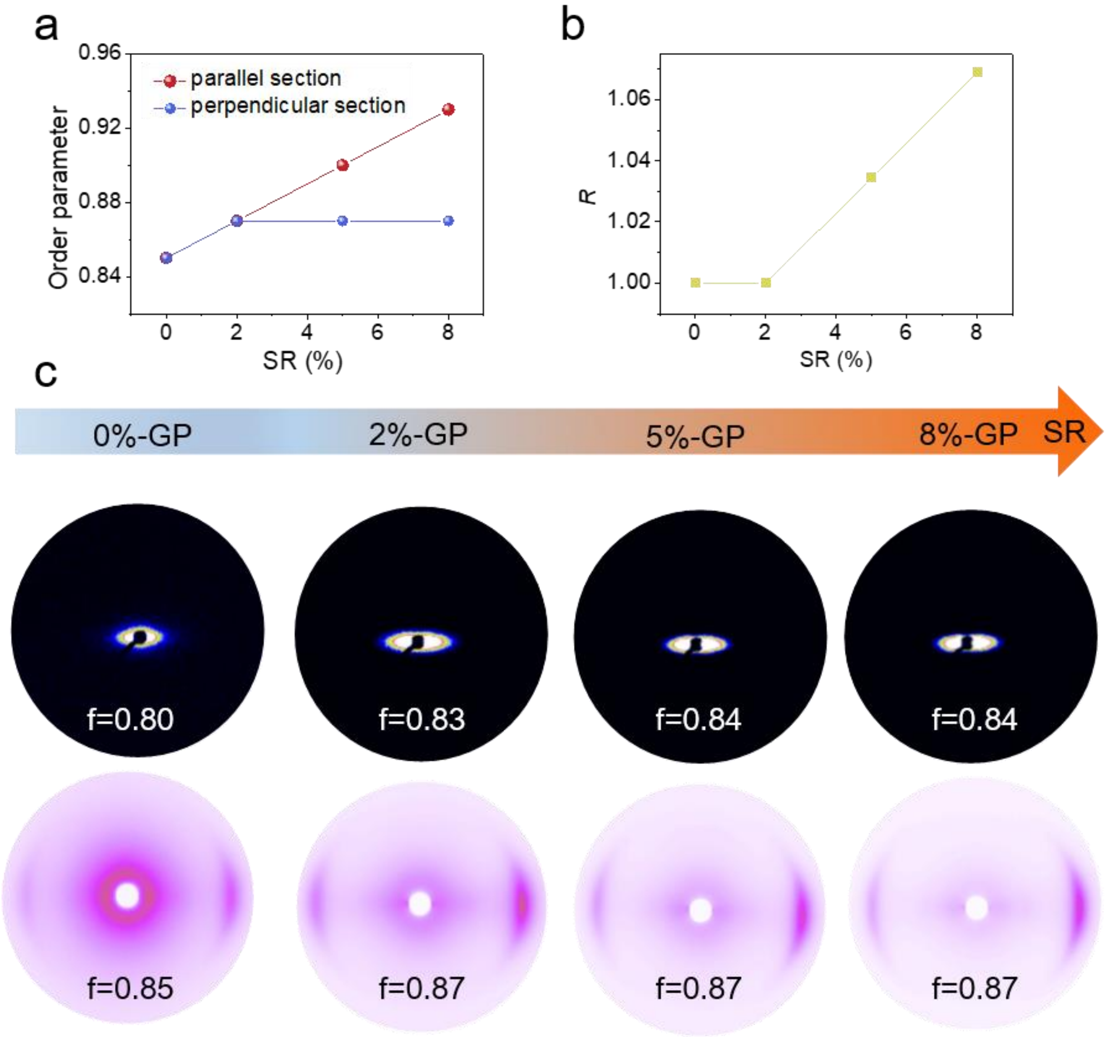

Supplementary Figure 8. The order parameter of graphene papers at different stretching ratio in perpendicular section in both nano-scale and atomic-scale. **a** The sheet order parameter of GPs at different *SR* in parallel and perpendicular sections. **b** Anisotropic ratio (R, $f_{//}$ / $f_{\perp}$) of GPs at different *SR*. **c** SAXS and WAXS patterns of GPs in perpendicular section.

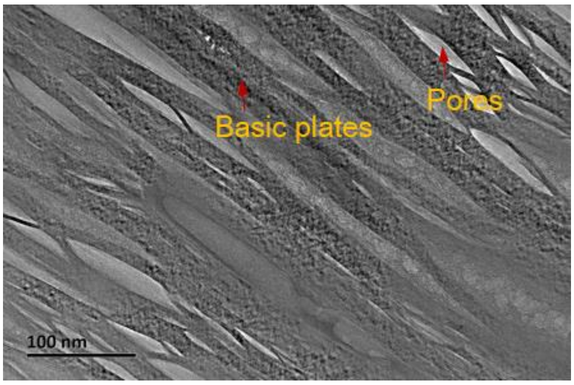

Supplementary Figure 9. Hierarchical structures of graphene papers, including basic plates and pores. Graphene sheets stack to form a basic plate with nanometers thickness and these plates pile up to become papers with micrometers thickness. Pores exist among these basic plates.

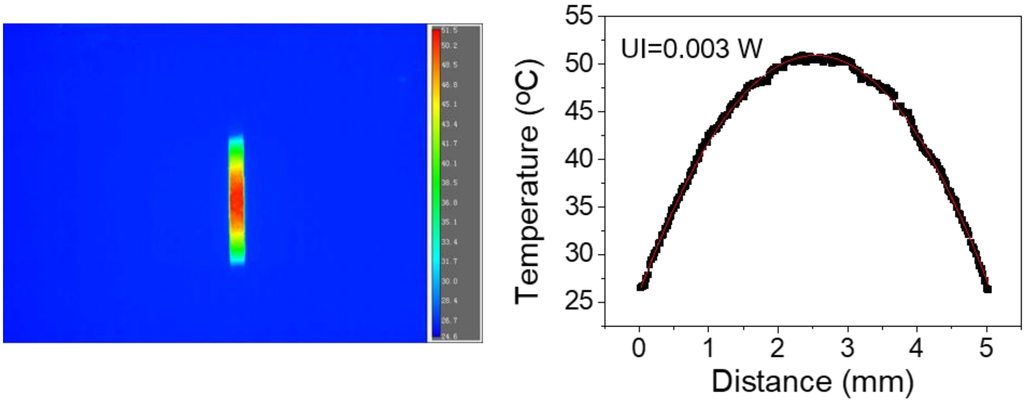

Supplementary Figure 10. The measurement of thermal conductivity of GP with stretching ratio of 8%.

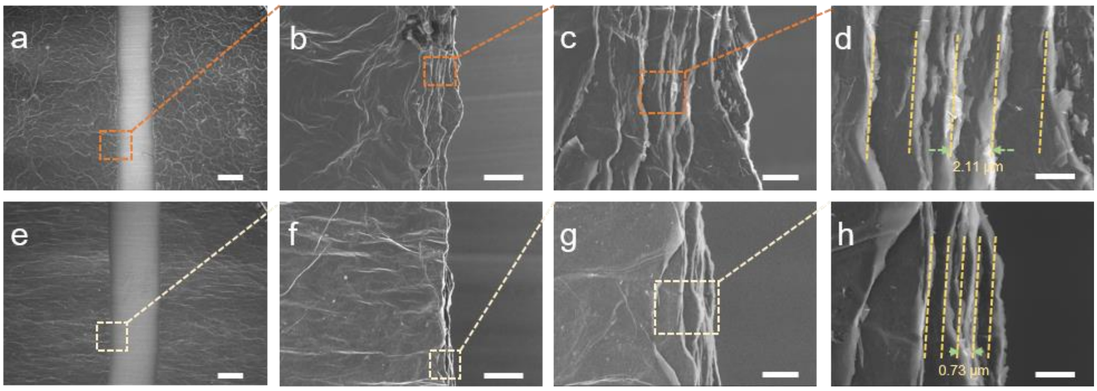

Supplementary Figure 11. The fracture morphology of graphene papers. The fracture of direct-cast GPs (a-d) and 8%-GPs (e-h). Lower slippage band depicts more brittle behavior of GPs. 8%-GPs show slippage band of only 0.73 μm, depicting that 8%-GPs after IMP stretching show considerably brittle with less slippage band width. Scale bar, 200 μm (**a, e**), 20 μm (**b, f**), 5 μm (**c, g**), and 2 μm (**d, h**)

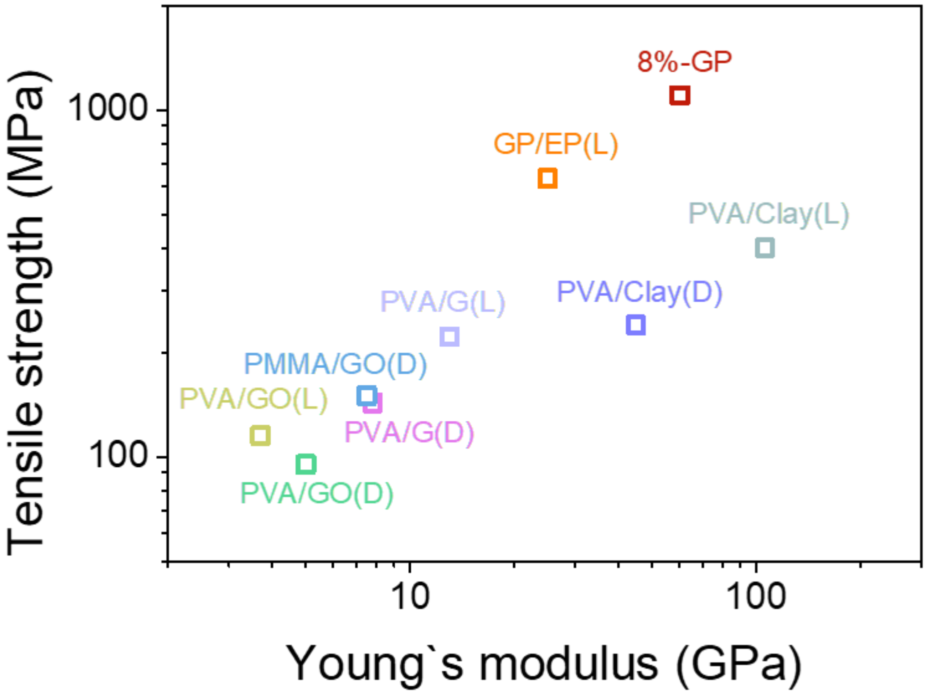

Supplementary Figure 12. Comparison of tensile strength and Young`s modulus for two-dimensional nanosheets-based composites[2-6], which were prepared by casting/coating from dispersions (labeled as D) and layer-by-layer assembly (labeled by L).

Reference	Strength (MPa)	Young`s modulus (GPa)	Preparation	Additive interlayer interaction
8%-GP	1100	60.27	Continuous IMP stretching; Solid processing	No
Direct-cast GP	234.16	7.6	Cast; Solution state	No
7	100	32	Vacuum-assisted filtration; Solution state	No
8	160	66.67	Vacuum-assisted filtration; Solution state	Borate crosslinking
9	178.96	84.84	Vacuum-assisted filtration; Solution state	PEI crosslinking
10	944	15.134	Vacuum-assisted filtration; Solution state	π-π and covalent crosslinking
11	300	42.3	Vacuum-assisted filtration; Solution state	No
12	129.6	1.96	Vacuum-assisted filtration; Solution state	π conjugated crosslinking
13	765	12.299	Cast; Solution state	CNC; Topological design
14	382	8.863	Cast; Solution state	No
15	614	10.406	Cast; Solution state	No
16	660	20	Centrifugal spraying; Solution state	No
17	606	43.1	Vacuum-assisted filtration; Solution state	No

Supplementary Table 1. Comparison of the graphene papers prepared by different methods. Our continuous IMP stretching method supplements the structural control of GPs in a solid plastic state, which completes the whole process of GPs from liquid dispersions to the final solid.

Supplementary References

1. Zhang, C., Fujii, Y. & Tanaka, K. Effect of long range interactions on the glass transition temperature of thin polystyrene films. *ACS Macro Lett.* **1**, 1317-1320 (2012).
2. Podsiadlo, P. *et al*. Ultrastrong and stiff layered polymer nanocomposites. *Science* **318**, 80-83 (2007).
3. Zhu, J., Zhang, H. & Kotov, N. A. Thermodynamic and structural insights into nanocomposites engineering by comparing two materials assembly techniques for graphene. *ACS Nano* **7**, 4818-4829 (2013).
4. Walther, A. *et al*. Large-area, lightweight and thick biomimetic composites with superior material properties via fast, economic, and green pathways. *Nano lett.* **10**, 2742-2748 (2010).
5. Liu, S. et al. Artificial bicontinuous laminate synergistically reinforces and toughens dilute graphene composites. *ACS Nano* **12**, 11235-11243 (2018).
6. Putz, K. W., Compton, O. C., Palmeri, M. J., Nguyer, S. T. & Brinson L. C. High-nanofiller-content graphene oxide-polymer nanocomposites via vacuum-assisted self-assembly. *Adv. Func. Mater.* **20**, 3322-3329 (2010).
7. Dikin, D. A. *et al.* Preparation and characterization of graphene oxide paper. *Nature* **448**, 457-460 (2007).
8. An, Z., Compton, O. C., Putz, K. W., Brinson, L. C. & Nguyen, S. T. Bio-inspired borate cross-linking in ultra-stiff graphene oxide thin films. *Adv. Mater.* **23**, 3842-3846 (2011).
9. Tian, Y., Cao, Y., Wang, Y., Yang, W. & Feng, J. Realizing ultrahigh modulus and high strength of macroscopic graphene oxide papers through crosslinking of mussel-inspired polymers. *Adv. Mater.* **25**, 2980-2983 (2013).
10. Wan, S. *et al.* Sequentially bridged graphene sheets with high strength, toughness, and electrical conductivity. *Proc. Natl. Acad. Sci. USA* **115**, 5359-5364 (2018).
11. Chen, H., Muller, M. B., Gilmore, K. J., Wallace, G. G. & Li, D. Mechanically strong, electrically conductive, and biocompatible graphene paper. *Adv. Mater.* **20**, 3557-3561 (2008).
12. Cheng, Q., Wu, M., Li, M., Jiang, L. & Tang, Z. Ultratough artificial nacre based on conjugated cross-linked graphene oxide. *Angew. Chem. Int. Ed.* **52**, 3750-3755 (2013).
13. Wen, Y., Wu, M., Zhang, M., Li, C. & Shi, G. Topological design of ultrastrong and highly conductive graphene films. *Adv. Mater.* **29**, 1702831 (2017).

14. Zhang, M., Huang, L., Chen, J., Li, C. & Shi, G. Ultratough, ultrastrong, and highly conductive graphene films with arbitrary sizes. *Adv. Mater.* **26**, 7588-7592 (2014).
15. Zhang, M. *et al*. Multifunctional pristine chemically modified graphene films as strong as stainless steel. *Adv. Mater.* **27**, 6708-6713 (2015).
16. Zhong, J. *et al*. Efficient and scalable synthesis of highly aligned and compact two-dimensional nanosheet films with record performances. *Nat. Commun.* **9**, 3484 (2018).
17. Wu, M. *et al*. Chemical approach to ultrastiff, strong, and environmentally stable graphene films. *ACS Appl. Mater. Interfaces* **10**, 5812-5818 (2018).
